# Supplementary material for: Genome-wide association study of placental weight identifies distinct and shared genetic influences between placental and fetal growth
Source: Nat Genet. 2023 Oct 5;55(11):1807–19. doi: 10.1038/s41588-023-01520-w (PMC10632150; doi:10.1038/s41588-023-01520-w)
Supplement: Supplementary file 2 — Reporting Summary [file 41588_2023_1520_MOESM2_ESM.pdf]

nature research

Corresponding author(s): Pål R. NJØLSTAD, BJARKE FEENSTRA, RACHEL M. FREATHY, STEFAN JOHANSSON

Last updated by author(s): Jul 7, 2023

Reporting Summary

Nature Research wishes to improve the reproducibility of the work that we publish. This form provides structure for consistency and transparency in reporting. For further information on Nature Research policies, see our Editorial Policies and the Editorial Policy Checklist.

Statistics

For all statistical analyses, confirm that the following items are present in the figure legend, table legend, main text, or Methods section.

n/a	Confirmed	
☐	☒	The exact sample size (*n*) for each experimental group/condition, given as a discrete number and unit of measurement
☐	☒	A statement on whether measurements were taken from distinct samples or whether the same sample was measured repeatedly
☐	☒	The statistical test(s) used AND whether they are one- or two-sided<br>*Only common tests should be described solely by name; describe more complex techniques in the Methods section.*
☐	☒	A description of all covariates tested
☐	☒	A description of any assumptions or corrections, such as tests of normality and adjustment for multiple comparisons
☐	☒	A full description of the statistical parameters including central tendency (e.g. means) or other basic estimates (e.g. regression coefficient) AND variation (e.g. standard deviation) or associated estimates of uncertainty (e.g. confidence intervals)
☐	☒	For null hypothesis testing, the test statistic (e.g. *F*, *t*, *r*) with confidence intervals, effect sizes, degrees of freedom and *P* value noted<br>*Give P values as exact values whenever suitable.*
☐	☒	For Bayesian analysis, information on the choice of priors and Markov chain Monte Carlo settings
☐	☒	For hierarchical and complex designs, identification of the appropriate level for tests and full reporting of outcomes
☐	☒	Estimates of effect sizes (e.g. Cohen's *d*, Pearson's *r*), indicating how they were calculated

Our web collection on statistics for biologists contains articles on many of the points above.

Software and code

Policy information about availability of computer code

Data collection	Data collection was conducted by the cohorts participating in the study, software and code used is documented in the cohort study publications.
Data analysis	The software and version used for the GWAS in the different cohorts is listed in Supplementary Tables 2, 4, and 6.<br>The code used to conduct the follow-up analyses is available at github.com/EarlyGrowthGenetics/placental_weight_code<br>Briefly:<br>- Meta-analysis was conducted using Metal (version dated 2011-03-25).<br>- Conditional and joint analysis was conducted using GCTA-COJO v1.26.0<br>- Colocation analyses were conducted using the R v4.0.0 package coloc v4.0-4<br>- Structural equation modeling was conducted using genomic SEM v0.0.5<br>- Trio and transmission analyses were conducted using WLM (script in repository) for the meta-analysis results and using TrioGen (https://github.com/mvaudel/trioGen v. 0.5.0)<br>- Placental meQTLs were identified using TensorQTL<br>- Polygenic scores were built using LD-pred2<br>- Phasing was conducted using SHAPEIT, SHAPEIT2, SHAPEIT3, PBWT, Eagle v2.3, Eagle v2.4, or IMPUTE2<br>- Imputation was conducted using IMPUTE2, IMPUTE3, Minimac3, Minimac4, PBWT, or the Sanger imputation server<br>- GWAS lookups were preformed using Phenoscanner v1.0<br><br>Custom analysis code is available in https://github.com/EarlyGrowthGenetics/placental_weight_code

For manuscripts utilizing custom algorithms or software that are central to the research but not yet described in published literature, software must be made available to editors and reviewers. We strongly encourage code deposition in a community repository (e.g. GitHub). See the Nature Research guidelines for submitting code & software for further information.

April 2020

Data

Policy information about availability of data

All manuscripts must include a data availability statement. This statement should provide the following information, where applicable:

- Accession codes, unique identifiers, or web links for publicly available datasets
- A list of figures that have associated raw data
- A description of any restrictions on data availability

GWAS summary statistics will be shared upon publication of the article at http://egg-consortium.org/placental-weight-2023.html.

Field-specific reporting

Please select the one below that is the best fit for your research. If you are not sure, read the appropriate sections before making your selection.

☒ Life sciences ☐ Behavioural & social sciences ☐ Ecological, evolutionary & environmental sciences

For a reference copy of the document with all sections, see nature.com/documents/nr-reporting-summary-flat.pdf

Life sciences study design

All studies must disclose on these points even when the disclosure is negative.

Sample size	In a GWAS meta-analysis, the more samples included, the more GWAS-significant loci will be detected.
Data exclusions	Gestational week lower than 37 weeks and over 42 weeks
Replication	This study is a meta-analysis. The results have not been replicated in external studies.
Randomization	This study is observational, so randomisation is not applicable to this type of study.
Blinding	This study is observational, so blinding is not applicable to this type of study.

Reporting for specific materials, systems and methods

We require information from authors about some types of materials, experimental systems and methods used in many studies. Here, indicate whether each material, system or method listed is relevant to your study. If you are not sure if a list item applies to your research, read the appropriate section before selecting a response.

Materials & experimental systems

n/a	Involved in the study	
☒	☐	Antibodies
☒	☐	Eukaryotic cell lines
☒	☐	Palaeontology and archaeology
☒	☐	Animals and other organisms
☒	☐	Human research participants
☒	☐	Clinical data
☒	☐	Dual use research of concern

Methods

n/a	Involved in the study	
☒	☐	ChIP-seq
☒	☐	Flow cytometry
☒	☐	MRI-based neuroimaging
